# Association of neighborhood physical activity facilities with incident cardiovascular disease

**DOI:** 10.1186/s12942-023-00340-9

**Published:** 2023-07-29

**Authors:** Yulin Huang, Huimin Zhao, Qiuju Deng, Yue Qi, Jiayi Sun, Miao Wang, Jie Chang, Piaopiao Hu, Yuwei Su, Ying Long, Jing Liu

**Affiliations:** 1 Center for Clinical and Epidemiologic Research, Beijing Anzhen Hospital, Capital Medical University, 100029 Beijing, China; 2 Beijing Institute of Heart, Lung and Blood Vessel Diseases, Beijing, 100029 China; 3 National Clinical Research Center of Cardiovascular Diseases, 100029 Beijing, China; 4 The Key Laboratory of Remodeling-Related Cardiovascular Diseases, Ministry of Education, Beijing, 100029 China; 5 The Beijing Municipal Key Laboratory of Clinical Epidemiology, 100029 Beijing, China; 6 School of Architecture and Hang Lung Center for Real Estate, Key Laboratory of Eco Planning & Green Building, Ministry of Education, Tsinghua University, Beijing, 100084 China; 7 School of Urban Design, Wuhan University, Wuhan, 430072 China

**Keywords:** Neighborhood environment, Physical activity, Cardiovascular disease, Incidence, Cohort study

## Abstract

**Background:**

The availability of physical activity (PA) facilities in neighborhoods is hypothesized to influence cardiovascular disease (CVD), but evidence from individual-level long-term cohort studies is limited. We aimed to assess the association between neighborhood exposure to PA facilities and CVD incidence.

**Methods:**

A total of 4658 participants from the Chinese Multi-provincial Cohort Study without CVD at baseline (2007–2008) were followed for the incidence of CVD, coronary heart disease (CHD), and stroke. Availability of PA facilities was defined as both the presence and the density of PA facilities within a 500-m buffer zone around the participants’ residential addresses. Time-dependent Cox regression models were performed to estimate the associations between the availability of PA facilities and risks of incident CVD, CHD, and stroke.

**Results:**

During a median follow-up of 12.1 years, there were 518 CVD events, 188 CHD events, and 355 stroke events. Analyses with the presence indicator revealed significantly lower risks of CVD (hazard ratio [HR] 0.80, 95% confidence interval ([CI] 0.65–0.99) and stroke (HR 0.76, 95% CI 0.60–0.97) in participants with PA facilities in the 500-m buffer zone compared with participants with no nearby facilities in fully adjusted models. In analyses with the density indicator, exposure to 2 and ≥ 3 PA facilities was associated with 35% (HR 0.65, 95% CI 0.47–0.91) and 28% (HR 0.72, 95% CI 0.56–0.92) lower risks of CVD and 40% (HR 0.60, 95% CI 0.40–0.90) and 38% (HR 0.62, 95% CI 0.46–0.84) lower risks of stroke compared with those without any PA facilities in 500-m buffer, respectively. Effect modifications between presence of PA facilities and a history of hypertension for incident stroke (*P* = 0.049), and a history of diabetes for incident CVD (*P* = 0.013) and stroke (*P* = 0.009) were noted.

**Conclusions:**

Residing in neighborhoods with better availability of PA facilities was associated with a lower risk of incident CVD. Urban planning intervention policies that increase the availability of PA facilities could contribute to CVD prevention.

**Supplementary Information:**

The online version contains supplementary material available at 10.1186/s12942-023-00340-9.

## Background

Cardiovascular disease (CVD) is the leading cause of death in China, accounting for 40% of deaths in the population [1]. A positive effect of physical activity (PA) on CVD was demonstrated in previous studies [2, 3], and regular PA is recommended to prevent CVD development [4]. It is believed that neighborhood facilities are essential to promote PA in the general population [5, 6]. However, the association between the availability of PA facilities and CVD risk remains unestablished.

A meta-analysis found that neighborhood environmental attributes that promote PA were associated with reductions in several CVD risk factors, including hypertension, diabetes, and obesity [7]. Moreover, the relationship between the availability of PA facilities and CVD was investigated in several studies in developed western countries, many of which were cross-sectional studies on the prevalence of CVD [8, 9] or the cardiovascular health score [10–12]. A few cohort studies investigated the association of PA facilities with the risk of CVD incidence. However, most of them determined the exposure to PA facilities based on area-level measurements or approximate residential locations using grid cells [13–15], rather than assessment on individuals’ residential addresses [16]. In addition, few cohort studies adjusted for individual-level traditional CVD risk factors [16].

To test the hypothesis that people living in neighborhoods with better availability of PA facilities are more likely to have a decreased risk of incident CVD, we examined the association between the availability of PA facilities and incidence of CVD using 12-year follow-up data in a population-based prospective cohort study.

## Methods

### Study population

The Chinese Multi-provincial Cohort Study (CMCS) is an ongoing, prospective, population-based cohort study [17, 18]. Initially, 16,811 participants aged 35–64 years were recruited from 16 centers in 11 provinces/province-level regions of China from 1992 to 1993 using a multistage sampling method. First, the centers were selected nonrandomly with the major requirements of having taken part in the Sino-Monitoring Trends and Determinants in Cardiovascular Disease (Sino-MONICA) Project [19] and being able to conduct the study. Among these centers, 12 centers were in urban areas and 4 centers were in rural areas. Then, a stratified random sampling was performed for each gender and age group (35–44, 45–54, and 55–64 years). In 2007–2008, 5961 individuals from seven centers in six provinces/ province-level regions participated in the re-examination. Due to the availability of data on PA facilities, participants from six urban centers of five provinces/province-level regions (Beida center and Shougang center in Beijing, Zhongshan center in Shanghai, Shenyang Angang center in Liaoning, Chengdu Huaxi center in Sichuan, and Daqing center in Heilongjiang) were enrolled in the study. Eventually, 4658 participants were included in this analysis after excluding individuals with established CVD at baseline (n = 404), missing baseline covariate data (n = 158), or identifiable residential addresses (n = 120) (See Additional file 1: Figure S1).

The study was approved by the Ethics Committee of Beijing Anzhen Hospital, Capital Medical University. Written informed consent was obtained from all participants. All research adhered to the tenets of the Declaration of Helsinki.

### Exposure assessment

To identify neighborhood PA facilities, we used point of interest (POI) data on the open platform of AutoNavi Map API in 2010. The data included basic information for each POI, such as code, location name, address, latitude, longitude, brand, and business categories, with latitude and longitude being the core attributes of the POI data. The three-level classification standard for a POI in AutoNavi Map was adopted for data classification. In the database, all PA facilities were objectively registered under different categories. As PA facilities, PA/recreational establishments were considered, including gyms, fitness centers, swimming pools, skating rinks, golf courses, and skiing facilities.

We geocoded the residential address of each participant at baseline into spatial coordinates and imported these coordinates into the geographic information system software. A 500-m buffer zone was calculated around each individual’s residence, as it is within the walking distance [20]. Availability of PA facilities, defined as both the presence and the density of PA facilities, was measured within the 500-m buffer zone. The presence of PA facilities was defined as having at least one PA facility in the buffer zone, while the density of PA facilities was defined as the total number of PA facilities within the buffer zone. A 1000-m buffer zone, which was used in previous research [21], was also used for sensitivity analysis for the density of PA facilities, but not for the presence indicator, because only a few participants (*n* = 266) had no PA facilities within a 1000-m buffer zone.

### Covariates

Demographic characteristics, smoking status, alcohol consumption, medical history, and medication history were collected using a standardized questionnaire. Individual-level demographic characteristics included age, sex, education (college or above vs. below college), occupation (intellectuals vs. non-intellectuals), and household monthly income per capita (0–3000 vs. > 3000 yuan). Intellectual occupations included professional or administrative staff, while non-intellectual occupations included workers, farmers, or unemployed. Smoking was defined as consumption of one or more cigarettes per day for at least 1 year. Current drinking was defined as alcohol consumption at least once a week. Height, weight, and blood pressure (BP) data were obtained by trained physicians during a physical examination. Body mass index (BMI) was calculated as weight in kilograms divided by height in meters squared. Overweight/obesity was defined as BMI ≥ 24 kg/m^2^ [22]. BP was measured three times after 5 min of rest using a calibrated sphygmomanometer, and the average of the second and third readings was recorded. Hypertension was defined as measured systolic BP ≥ 140 mm Hg and/or measured diastolic BP ≥ 90 mm Hg or receipt of antihypertensive medications within 2 weeks. Diabetes was defined as fasting blood glucose ≥ 7.0 mmol/L or previous physician diagnosis. Venous blood samples were collected after at least 8 h of fasting. Levels of fasting blood glucose (FBG) and total cholesterol (TC) were measured on the sample collection day as described previously [18]. Township-level population density for each individual’s residence was determined as the number of people per square kilometer. The township is the smallest administrative unit in China. The means of area and population size of the townships where the study population located were 31.0 km^2^, and 132,493 persons. The distance to the major road was measured by the Euclidean distance from each participant’s residential address to the major road [23]. Leisure-time PA was recorded as four categories: no PA, mild PA, moderate or high intensity PA for > 30 min on 1–2 days per week, and moderate or high intensity PA for > 30 min on 3 or more days per week in leisure time. In current analyses, we combined the top two categories with moderate or high intensity leisure-time PA for > 30 min at least once a week, and converted leisure-time PA into a binary variable (yes or no).

### Outcome assessment

All participants were followed up for incident CVD, coronary heart disease (CHD), and stroke every 1–2 years by active interviews, supplemented by linkage to local vital registration systems and hospital information systems. Acute CHD events included non-fatal acute myocardial infarction and all coronary deaths. Acute stroke events included subarachnoid hemorrhage, intracerebral hemorrhage, or cerebral infarction. Diagnostic criteria for CVD events were based on the World Health Organization-MONICA project and modified according to advances in diagnostic technology for myocardial infarction [18, 19, 24]. All reported events were adjudicated by a panel of physicians that included cardiologists and general physicians.

### Sample power estimation

The statistical power was estimated respectively based on the two indicators of availability of PA facilities in 500-m buffer.

(1) For the presence indicator of PA facilities in 500-m buffer.

In this study, presence of PA facilities was significantly associated with a 20% lower risk (HR = 0.80, 95%CI 0.65–0.99) of incident CVD compared with no nearby facilities. The number of CVD cases during the follow-up was 518. The proportion of participants exposed to PA facilities was 78.4%. Assessing an alpha (probability of type I error) of 0.05, the actual sample size of 4658 enabled a statistical power of 0.999.

(2) For the density indicator of PA facilities in 500-m buffer.

In our study, density of PA facilities ≥ 3 was significantly related to a decreased risk of incident CVD (HR = 0.72, 95% CI 0.56–0.92). With an alpha of 0.05 and the number of cases of 518, the actual sample size of 4658 provided a statistical power of 0.999.

### Statistical analysis

Continuous variables were presented as mean ± SD for normally distributed variables or median and interquartile range for non-normally distributed variables. Categorical variables were expressed as number and percentage. The baseline characteristics of the study participants were compared using the presence indicator for PA facilities in the 500-m buffer zone.

Time-dependent Cox proportional hazards regression models were performed to estimate the hazard ratio (HR) and 95% confidence interval (CI) of incident CVD with availability of PA facilities. The proportional hazards assumption was tested by evaluating the Schoenfeld residuals [25]. Multivariate analyses included adjustment for variable × follow-up time (log function of time) interactions to account for time-varying confounders that were against the proportional hazards assumption [26]. We entered the indicators for PA facilities as categorical variables by defining their categories based on either counts or presence and absence of PA facilities. The cut-off values of categorical variables for the density indicators within each level were set in a way that ensured sufficient numbers of individuals remained at each level. An unadjusted model (model 1) was performed first. In Model 2, we adjusted for possible confounders including sex, age, education, occupation, household income, smoking, current drinking, levels of BMI, systolic BP, FBG, and TC, use of antihypertensive, glucose-lowering, and statin drugs, and population density. In Model 3, we further adjusted for leisure-time PA. Subgroup analyses were performed for baseline demographic characteristics, traditional CVD risk factors, and leisure-time PA. The HRs between subgroups were compared using the Z-test [27]. We examined the mediation role of leisure-time PA on the association of the presence of PA facilities in 500-m buffer with CVD and stroke.

Additional sensitivity analyses were performed in this study as follows: (a) using the 1000-m buffer zone; (b) excluding participants who experienced events during the first year of follow-up within the 500-m buffer zone (n = 25); (c) further adjusting for distance to the major road from each participant’s residence based on the multivariate models (Model 3); (d) exploring the association of the availability of fitness centers (the main type of PA facilities) with CVD incidence; (e) using the shared frailty models.

Statistical analyses were performed using R software (version 4.1.1). Two-tailed values of *P* < 0.05 were considered to indicate statistical significance.

## Results

### Baseline characteristics of the study participants

The mean age of the participants (*n* = 4658) was 61.2 ± 7.6 years at baseline, and 2369 (50.9%) participants were women. PA facilities were present within the 500-m buffer zone around their residences for 3654 (78.4%) individuals. The baseline characteristics of the participants were compared according to the presence of PA facilities within the 500-m buffer zone. Participants exposed to PA facilities (≥ 1 PA facility in the buffer zone) were more likely to be older, have higher proportion of college education, have higher household monthly income, and have lower proportion of current drinking. Individuals with exposure to PA facilities had a significantly lower level of diastolic BP and higher proportions of hypercholesteremia, antihypertensive treatment, statin treatment, and leisure-time PA than those with no nearby PA facilities. The level of population density was higher among participants exposed to PA facilities (Table 1). Of all the PA facilities within the 1000-m buffer around residential addresses of participants, fitness centers accounted for the largest proportion (73.39%), followed in descending order by gyms (20.50%), swimming pools (3.07%), golf courses (2.55%), skating rinks (0.46%), and skiing facilities (0.03%).

**Table 1:** Baseline characteristics by presence of physical activity facilities within a 500-m buffer around residential addresses

	Presence of physical activity facilities		*P* value
	No	Yes	
N	1004	3654	
Age, mean ± SD, years	61.1 ± 7.1	61.2 ± 7.7	< 0.001
Sex, n (%)			0.742
Male	498 (49.6)	1791 (49.0)	
Female	506 (50.4)	1863 (51.0)	
Education, n (%)			< 0.001
Below college	852 (84.9)	2682 (73.4)	
College or above	152 (15.1)	972 (26.6)	
Occupation, n (%)			0.246
Intellectuals	618 (61.6)	2322 (63.5)	
Non-intellectuals	386 (38.4)	1332 (36.5)	
Household monthly income per capita, n (%)			< 0.001
0–3000 Yuan	895 (89.1)	2467 (67.5)	
> 3000 Yuan	109 (10.9)	1187 (32.5)	
Smoking, n (%)	199 (19.8)	643 (17.6)	0.105
Current drinking, n (%)	188 (18.7)	577 (15.8)	0.026
Leisure-time PA, n (%)	340 (33.9)	1515 (41.5)	< 0.001
SBP, mean ± SD, mmHg	137.0 ± 19.0	136.0 ± 18.8	0.530
DBP, mean ± SD, mmHg	82.5 ± 10.8	81.9 ± 10.2	0.047
FBG, mean ± SD, mmol/L	5.6 ± 1.6	5.7 ± 1.5	0.210
TC, mean ± SD, mmol/L	5.1 ± 1.0	5.2 ± 1.0	0.087
BMI, mean ± SD, kg/m^2^	24.8 ± 3.2	24.8 ± 3.3	0.439
Hypertension, n (%)	509 (50.7)	1957 (53.6)	0.108
Diabetes, n (%)	150 (14.9)	553 (15.1)	0.879
Hypercholesteremia, n (%)	118 (11.8)	582 (15.9)	0.001
Antihypertensive treatment, n (%)	241 (24.0)	1162 (31.8)	< 0.001
Glucose-lowering treatment, n (%)	87 (8.7)	301 (8.2)	0.664
Statin treatment, n (%)	14 (1.4)	111 (3.0)	0.004
Population density, median (interquartile range), persons/km^2^	1004 (224, 8214)	10,073 (2384, 15,123)	< 0.001

Data are expressed as number (percent) for categorical variables and as mean ± SD for continuous variables in case of normal distributions and median (interquartile range) otherwise

*P* values were calculated with unpaired Student’s t-test (when satisfying a normal distribution) or Mann–Whitney U test (when not satisfying a normal distribution) for continuous variables, and χ^2^ test for categorical variables

*BMI* body mass index, *DBP* diastolic blood pressure, *FBG* fasting blood glucose, *N* number of participants, *PA* physical activity, *SBP* systolic blood pressure, *TC* total cholesterol

### Association between the availability of PA facilities and risk of incident CVD

Over a median follow-up of 12.1 years (51,674.6 person-years), 518 incident CVD events, 188 CHD events, and 355 stroke events occurred among the participants. Table 2 shows the HRs for incident CVD, CHD, and stroke with presence of PA facilities in the 500-m buffer zone around the participants’ residences. The participants exposed to PA facilities within the buffer zone had significantly lower risks of CVD, CHD, and stroke in the univariate analyses (Model 1) compared with participants living with no PA facilities. After adjustment for demographic characteristics, traditional CVD risk factors, and population density (Model 2) and further adjustment for leisure-time PA (Model 3), the associations between the presence of PA facilities and CVD or stroke remained significant, with HR (95% CI) of 0.80 (0.65–0.99) for CVD and 0.76 (0.60–0.97) for stroke in model 3. However, the association between the presence of PA facilities and incident CHD did not reach significance, with HR (95% CI) of 0.80 (0.56–1.13) in the fully adjusted model (Table 2). The mediation analyses showed the mediating effect of leisure-time PA was not significant on the association between presence of PA facilities in 500-m buffer and CVD and stroke (See Additional file 1: Table S1).

**Table 2:** Association between presence of physical activity facilities within a 500-m buffer and incident CVD

	N	No. of cases	Model 1 HR (95% CI)	Model 2 HR (95% CI)	Model 3 HR (95% CI)
CVD					
No PA facilities	1004	150	Reference	Reference	Reference
Presence of PA facilities	3654	368	0.59 (0.48, 0.71)	0.79 (0.64, 0.97)	0.80 (0.65, 0.99)
CHD					
No PA facilities	1004	52	Reference	Reference	Reference
Presence of PA facilities	3654	136	0.62 (0.45, 0.85)	0.77 (0.54, 1.09)	0.80 (0.56, 1.13)
Stroke					
No PA facilities	1004	110	Reference	Reference	Reference
Presence of PA facilities	3654	245	0.54 (0.43, 0.67)	0.76 (0.59, 0.97)	0.76 (0.60, 0.97)

Model 1: not adjusted

Model 2: adjusted by age, sex, education, occupation, household income, smoking, current drinking, body mass index, systolic blood pressure, fasting blood glucose, total cholesterol, antihypertensive treatment, glucose-lowering treatment, statin treatment, and population density

Model 3: adjusted for covariates in Model 2 plus additional adjustment for leisure-time physical activity

*CHD* coronary heart disease, *CI* confidence interval, *CVD* cardiovascular disease, *HR* hazard ratio, *N* number of participants, *No.* number, *PA* physical activity

**Fig. 1:**
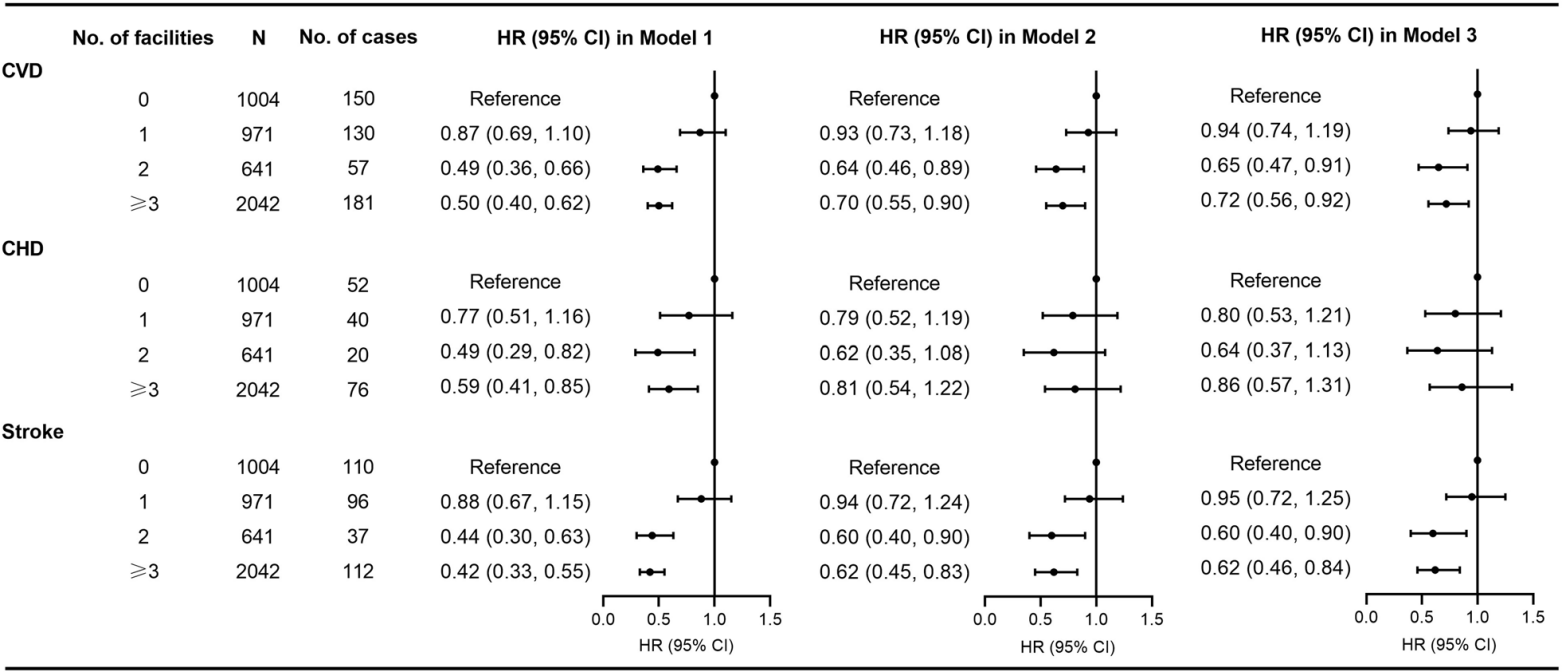
Association between density of physical activity facilities and incident CVD in 500-m buffer. Model 1: not adjusted. Model 2: adjusted by age, sex, education, occupation, household income, smoking, current drinking, body mass index, systolic blood pressure, fasting blood glucose, total cholesterol, antihypertensive treatment, glucose-lowering treatment, statin treatment, and population density. Model 3: adjusted for covariates in Model 2 plus additional adjustment for leisure-time physical activity. *CHD* coronary heart disease, *CI* confidence interval, *CVD* cardiovascular disease, *HR* hazard ratio, *N* number of participants, *No.* number

The association between the density of PA facilities in the 500-m buffer zone and incident CVD is presented in Fig. 1. Compared with participants with no PA facilities in 500-m buffer, participants with 2 and ≥ 3 facilities in the buffer zone had significantly lower risks of CVD and stroke, while significant associations were not found for participants with only 1 PA facility in the buffer zone. Overall, exposure to ≥ 2 facilities was associated with 30% lower risk of CVD (HR 0.70, 95% CI 0.55–0.89) and 39% lower risk of stroke (HR 0.61, 95% CI 0.46–0.82) compared with no PA facilities after full adjustment.

Similar results were found for the relationship between the density of PA facilities and incident CVD using the 1000-m buffer zone (See Additional file 1: Figure S2) and excluding participants who experienced events during the first year of follow-up in the 500-m buffer zone (See Additional file 1: Figure S3). The results accounting for additional adjustment for the distance to major road from each participant’s residential address (See Additional file 1: Table S2), and analyzing the association between availability of the main type of PA facilities and incident CVD were also consistent with our main findings (See Additional file 1: Table S3 and Figure S4). The associations between the availability of PA facilities and incident CVD were attenuated and became non-significant using shared frailty models (See Additional file 1: Table S4 and Figure S5).

### Subgroup analyses for the association between the presence of PA facilities and risk of incident CVD

**Fig. 2:**
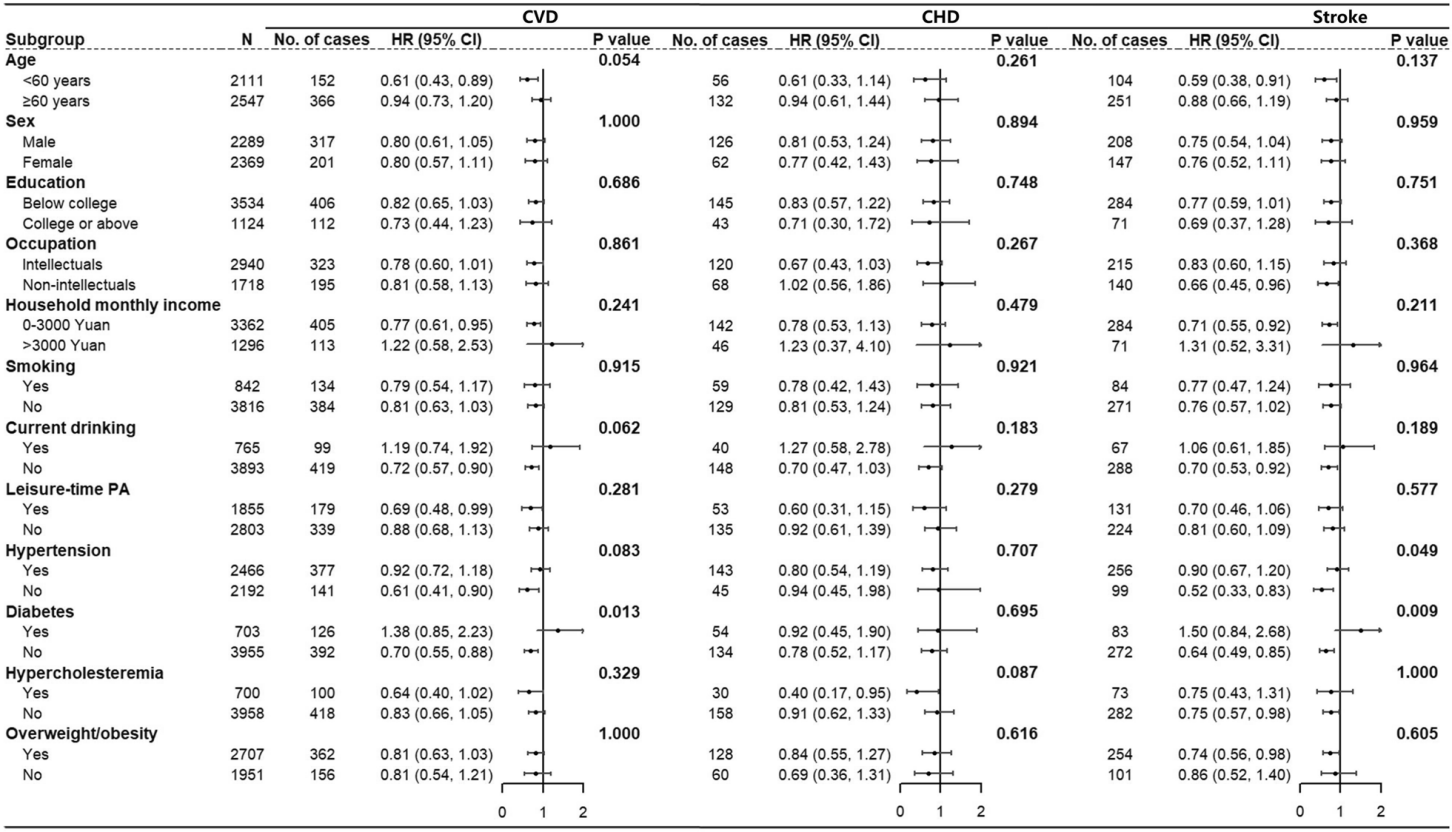
Subgroup analyses for the association between presence of physical activity facilities and CVD in 500-m buffer. All HRs were adjusted by age, sex, education, occupation, household income, smoking, current drinking, body mass index, systolic blood pressure, fasting blood glucose, total cholesterol, antihypertensive treatment, glucose-lowering treatment, statin treatment, population density and leisure-time physical activity, except for the grouping variable. *P*-values were obtained by analyzing the differences between the HRs derived from the subgroup analyses using the Z-statistic. *CHD* coronary heart disease, *CI* confidence interval, *CVD* cardiovascular disease, *HR* hazard ratio, *N* number of participants, *No.* number, *PA* physical activity. A horizontal arrow ( →) indicates that the 95% CI of the HR was outside the range of the values on the axis

The association between the presence of PA facilities within the 500-m buffer zone and risk of incident CVD was evaluated in various subgroup analyses. Except for significant effect modifications between the presence of PA facilities and a history of hypertension for incident stroke (*P* = 0.049), and a history of diabetes for incident CVD (*P* = 0.013), and stroke (*P* = 0.009), no other effect modifications were found. The negative association was diminished in participants with history of hypertension and diabetes (Fig. 2).

## Discussion

In this long-term population-based cohort study, participants living in urban areas with PA facilities, particularly ≥ 2 PA facilities, within a 500-m buffer zone of their residential addresses were found to have lower risks of incident CVD and stroke. Furthermore, the associations were independent of traditional CVD risk factors, socioeconomic factors, and leisure-time PA. These findings suggest that improvement in the availability of PA facilities around residences may promote CVD prevention.

Previous studies demonstrated that PA was protective against CVD and that greater neighborhood PA resources were associated with lower risks of CVD risk factors [28–30]. However, it remained unclear whether an increasing number of PA facilities was associated with a decreasing risk of CVD. The present findings for relationships between the availability of PA facilities and incident CVD are supported by some, but not all, previous cohort studies. In the Cardiovascular Health Study (CHS), higher density of neighborhood PA facilities in a 5-km buffer zone around residences was associated with a reduced risk of incident CVD in predominantly African American residential communities in the United States. However, the reduction in risk was not significant within a 1000-m buffer zone [16]. The present study confirmed this association in a population with a different ethnic background and neighborhood environment. There may be reliance on cars or public transportation to have access to PA resources in larger areas within the residential neighborhoods evaluated by the CHS in the United States, while the participants in the CMCS were expected to access their PA facilities by walking within a 500-m buffer zone. These findings suggested that people in different regions of the world may have different preferred travel distances to PA facilities depending on their car ownership, lifestyle, and other factors. However, we could not capture the actual utilization on neighborhood PA facilities of participants in this study. Further studies focused on the emerging technologies that could better quantify health-related behaviors, such as anonymized mobile phone location system, lifestyle sensors, wearable devices, and geo‑analytics and intelligence, are called for to provide more evidence for the association between availability of PA facilities and incident CVD [31, 32]. Meanwhile, a Swedish cohort study involving a nationwide sample found that availability of PA facilities was paradoxically associated with increased risks of stroke and CHD [13]. The associations were weakened for incident stroke and disappeared for incident CHD after adjusting for neighborhood-level and individual-level factors for socioeconomic status. Earlier studies showed that the risk of CVD was strongly influenced by socioeconomic characteristics, such as social disorganization (violent crime and unemployment) [33] and low social capital [34], besides the availability of neighborhood resources. Therefore, studies incorporating multiple relevant socioeconomic characteristics are needed to understand the apparently contradictory findings. Two further cohort studies using nationwide Swedish data [14, 15] found that PA facilities were not significantly associated with CHD or stroke after full adjustment for age, income, and neighborhood-level deprivation. However, data for lifestyle factors, medications, and laboratory measurements were not available in these Swedish studies, and residual confounding may have contributed to the findings.

The present findings showed that availability of PA facilities was significantly associated with incident stroke, but not with incident CHD. To a certain extent, these results were similar to the findings for associations between PA facilities and CVD in the nationwide Swedish sample, indicating that the associations between availability of resources and CVD were slightly stronger for stroke than for CHD [13]. Prior studies showed that the influence of genetics on the risk of stroke was weaker than that for other cardiovascular manifestations [35], suggesting that environmental factors, lifestyle factors, and other modifiable risk factors may have stronger influence on the development of stroke than on the development of CHD.

There have been some insights into potential mechanisms for the association between the availability of PA facilities and CVD. Better availability of PA resources was related to improvements in CVD risk factors, including reduced insulin resistance [28], decreased risk of type 2 diabetes [29], smaller waist circumference, lower BMI, and reduced body fat percentage [30]. In the present subgroup analyses, the association between PA facilities and CVD was significant in non-hypertensive and non-diabetic individuals but attenuated in those with history of hypertension and diabetes, suggesting that the effect of PA facilities may be more pronounced for primordial prevention. The relationship between availability of PA facilities and CVD remained largely unchanged after adjustment for leisure-time PA, and the mediating effect of leisure-time PA was not significant on the association between availability of PA facilities and CVD. One of the potential explanations is that individuals, particularly the older adults, may have other fitness options besides using the PA facilities. Previous studies about participation patterns for various PA types indicated that more prevalent activities among older adults tended to be walking, yard work/gardening and home exercises [36, 37]. Moreover, better availability of PA facilities may also be in contact with other beneficial neighborhood environmental conditions, such as better neighborhood socioeconomic status [38], together leading to health and well-being. This may partially explain the attenuation of association between availability of PA facilities and incident CVD after considering regional effect in the shared frailty models. Further studies are needed to explore the complicated mechanisms underlying the relationship between PA facilities and risk of CVD.

The study has several strengths. First, to our knowledge, this is the first cohort study to evaluate the association between the availability of PA facilities and risk of CVD in a non-western neighborhood context. Second, we objectively and simultaneously measured the presence and density indicators for availability of PA facilities at the individual-level. Finally, we adjusted for a wide range of individual-level risk factors for CVD and variables for socioeconomic status.

However, some limitations of the study should also be considered. First, there was a temporal mismatch between the residential geocoded information in the 2007–2008 period and the most recent datasets for the PA facilities (2010). However, the changes in the availability of PA facilities in this 2–3-year period are expected to be small. Furthermore, the participants who experienced events during the first year of follow-up were excluded from the sensitivity analyses, and similar results were obtained. Second, there was no available information on PA facility utilization by the participants (e.g., how often individuals went to the gym and how long they spent in the gym). Third, some potential environment confounders, such as transportation noise [39, 40] or air pollution [41], which have been demonstrated to be related to cardiovascular disease, were not adjusted for in this study. Previous studies showed the prevention to noise, especially from road or airport [42, 43] is important to avoid adverse effects on health outcomes such as learning impairment during early development [44], elevated diastolic blood pressure and hypertension [45, 46]. However, a previous study identified the distance to the major road from the sampling site as the top one contributor to predicting traffic noise [23]. Although the data of transportation noise was not available in our study, the sensitivity analyses additionally adjusting for the distance to major road from each participant’s residential address were conducted. The results showed better availability of PA facilities in 500-m buffer was significantly associated with a lower risk of CVD, which were consistent with our main findings. Moreover, we did not take account for the changes on PA facilities near residences during the study period in order to avoid reverse causality. In this analysis, we focused on the association between baseline availability of PA facilities near residences with the incidence of CVD events afterwards. Finally, the study did not account for any changes in residential addresses or levels of covariates, which are common limitations in long-term cohort studies.

## Conclusions

Living in areas with better availability of PA facilities was associated with a lower risk of incident CVD, independently of traditional CVD risk factors, socioeconomic status, and leisure-time PA. The present findings provide evidence for population-level interventions from the perspective of built environment to mitigate the incidence of CVD. Our findings require verification by prospective studies with other neighborhood characteristics, and the underlying mechanisms warrant further investigation.

## Supplementary Information

**Additional file 1: Figure S1.** Flow chart of study population. **Figure S2.** Association between density of physical activity facilities and incident CVD in 1000-m buffer. **Figure S3.** Association between density of physical activity facilities and incident CVD in 500-m buffer after excluding participants who occurred events in the first year of follow-up. **Figure S4.** Association between density of fitness centers and incident CVD in 500-m buffer. **Figure S5.** Association between density of physical activity facilities in 500-m buffer and incident CVD using shared frailty models. **Table S1.** Mediation analyses of the association between the presence of physical activity facilities in 500-m buffer and incident CVD through leisure-time physical activity. **Table S2.** Association between the availability of physical activity facilities in 500-m buffer and incident CVD after additionally adjusting for distance to the major road from each participant’s residence. **Table S3.** Association between presence of fitness centers in 500-m buffer and incident CVD. **Table S4.** Association between presence of physical activity facilities in 500-m buffer and incident CVD using shared frailty models.

## Data Availability

The datasets generated and/or analyzed during the current study are not publicly available due to ethical constraints in consideration of participants’ privacy and intellectual property protection, but are available from the corresponding author on reasonable request.

## Abbreviations

BMI: Body mass index
BP: Blood pressure
CHD: Coronary heart disease
CHS: Cardiovascular Health Study
CI: Confidence interval
CMCS: Chinese Multi-provincial Cohort Study
CVD: Cardiovascular disease
FBG: Fasting blood glucose
HR: Hazard ratio
MONICA: Monitoring Trends and Determinants in Cardiovascular Disease
PA: Physical activity
POI: Point of interest
TC: Total cholesterol
