# Supplementary material for: Association of neighborhood physical activity facilities with incident cardiovascular disease
Source: Int J Health Geogr. 2023 Jul 29;22:16. doi: 10.1186/s12942-023-00340-9 (PMC10386722; doi:10.1186/s12942-023-00340-9)
Supplement: Supplementary file 1 — Additional file 1: Figure S1. Flow chart of study population. Figure S2. Association between density of physical activity facilities and incident CVD in 1000-m buffer. Figure S3. Association between density of physical activity facilities and incident CVD in 500-m buffer after excluding participants who occurred events in the first year of follow-up. Figure S4. Association between density of fitness centers and incident CVD in 500-m buffer. Figure S5. Association between density of physical activity facilities in 500-m buffer and incident CVD using shared frailty models. Table S1. Mediation analyses of the association between the presence of physical activity facilities in 500-m buffer and incident CVD through leisure-time physical activity. Table S2. Association between the availability of physical activity facilities in 500-m buffer and incident CVD after additionally adjusting for distance to the major road from each participant’s residence. Table S3. Association between presence of fitness centers in 500-m buffer and incident CVD. Table S4. Association between presence of physical activity facilities in 500-m buffer and incident CVD using shared frailty models. [file 12942_2023_340_MOESM1_ESM.docx]

**Association of Neighborhood Physical Activity Facilities with Incident Cardiovascular Disease**

**Additional file Appendix**

1. **Additional file Figures**

- Additional file Figure 1. Flow chart of study population.
- Additional file Figure 2. Association between density of physical activity facilities and incident CVD in 1000-m buffer.
- Additional file Figure 3. Association between density of physical activity facilities and incident CVD in 500-m buffer after excluding participants who occurred events in the first year of follow-up.
- Additional file Figure 4. Association between density of fitness centers and incident CVD in 500-m buffer.
- Additional file Figure 5. Association between density of physical activity facilities in 500-m buffer and incident CVD using shared frailty models.

1. **Additional file Tables**

- Additional file Table 1. Mediation analyses of the association between the presence of physical activity facilities in 500-m buffer and incident CVD through leisure-time physical activity.
- Additional file Table 2. Association between the availability of physical activity facilities in 500-m buffer and incident CVD after additionally adjusting for distance to the major road from each participant’s residence.
- Additional file Table 3. Association between presence of fitness centers in 500-m buffer and incident CVD.
- Additional file Table 4. Association between presence of physical activity facilities in 500-m buffer and incident CVD using shared frailty models.

1. **Additional file Figures**


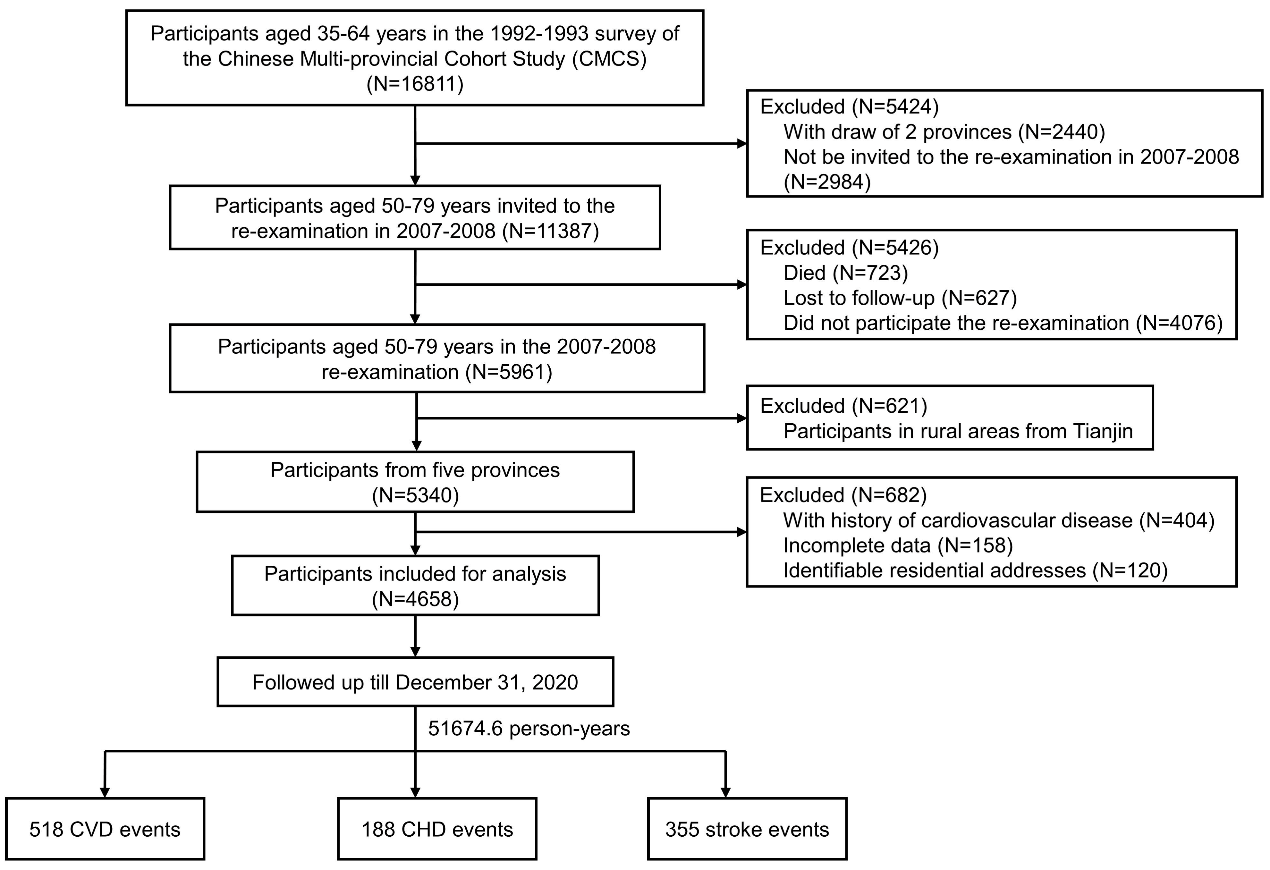


Additional file Figure 1. Flow chart of study population.

Abbreviations: CHD, coronary heart disease; CVD, cardiovascular disease.


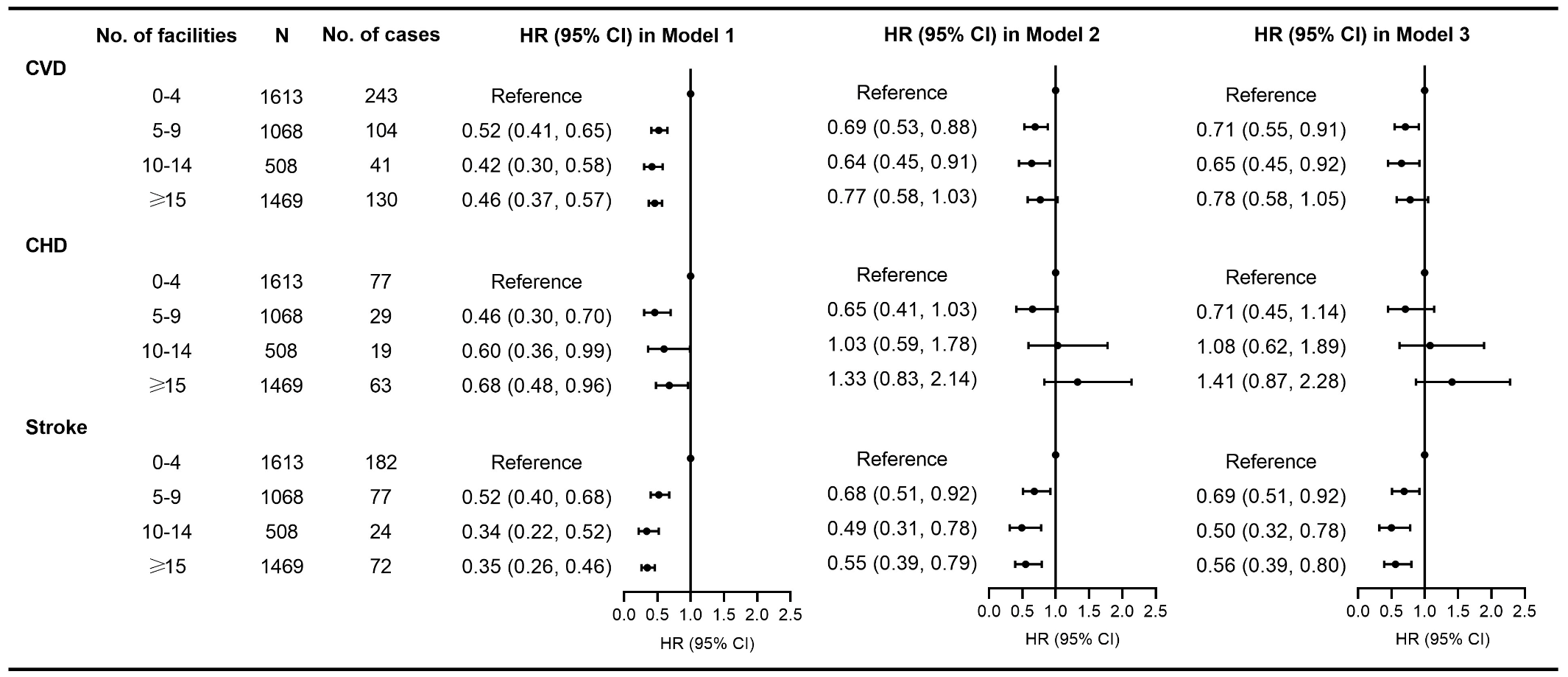


Additional file Figure 2. Association between density of physical activity facilities and incident CVD in 1000-m buffer.

Model 1: not adjusted.

Model 2: adjusted by age, sex, education, occupation, household income, smoking, current drinking, body mass index, systolic blood pressure, fasting blood glucose, total cholesterol, antihypertensive treatment, glucose-lowering treatment, statin treatment, and population density.

Model 3: adjusted for covariates in Model 2 plus additional adjustment for leisure-time physical activity.

Abbreviations: CHD, coronary heart disease; CI, confidence interval; CVD, cardiovascular disease; HR, hazard ratio; N, number of participants; No., number.


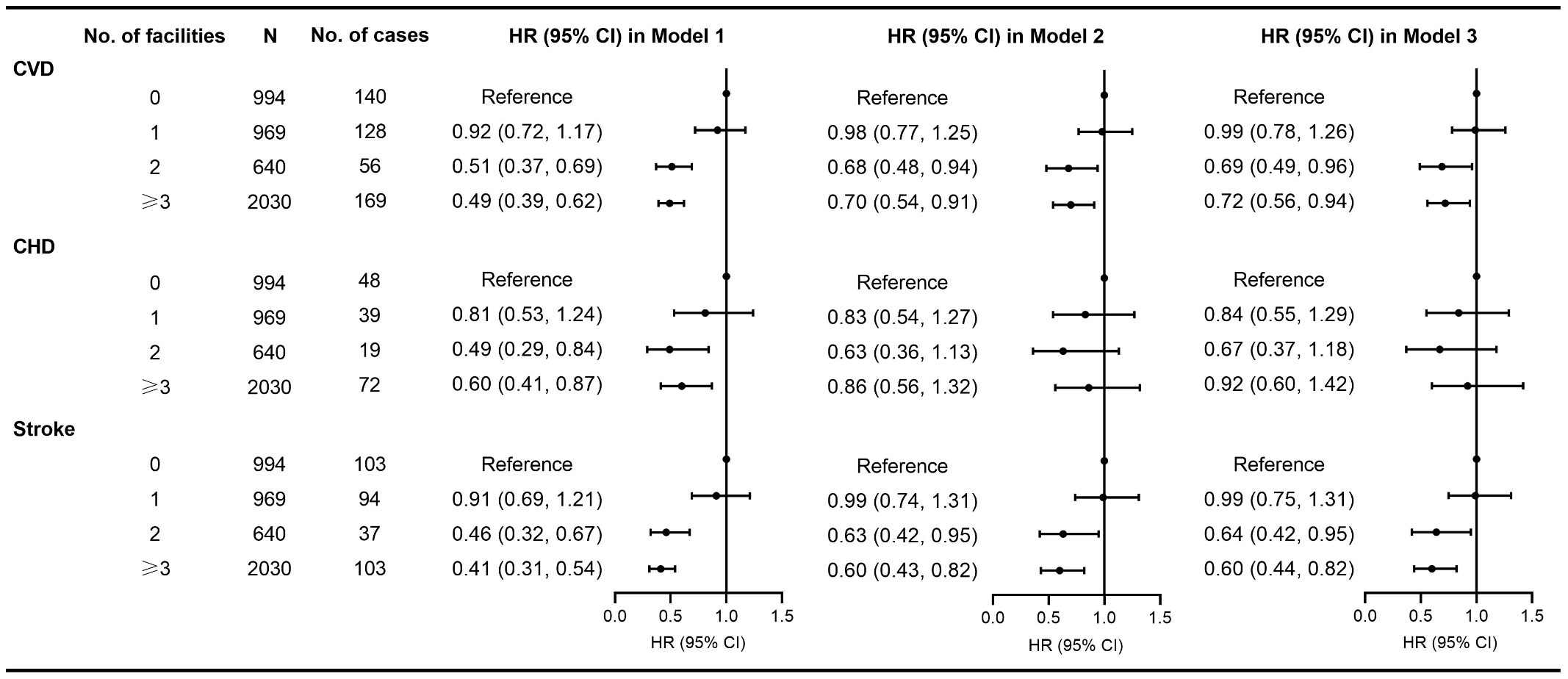


Additional file Figure 3. Association between density of physical activity facilities and incident CVD in 500-m buffer after excluding participants who occurred events in the first year of follow-up.

Model 1: not adjusted.

Model 2: adjusted by age, sex, education, occupation, household income, smoking, current drinking, body mass index, systolic blood pressure, fasting blood glucose, total cholesterol, antihypertensive treatment, glucose-lowering treatment, statin treatment, and population density.

Model 3: adjusted for covariates in Model 2 plus additional adjustment for leisure-time physical activity.

Abbreviations: CHD, coronary heart disease; CI, confidence interval; CVD, cardiovascular disease; HR, hazard ratio; N, number of participants; No., number.


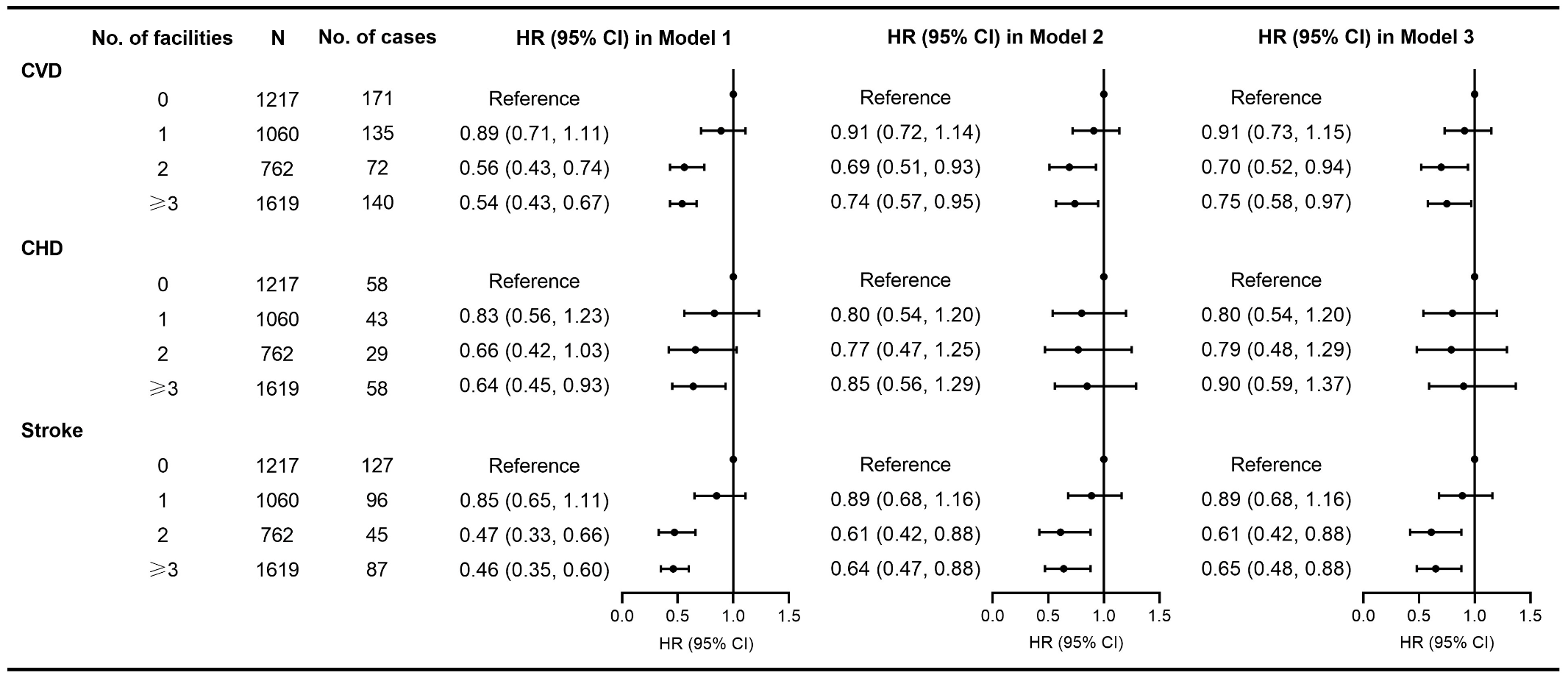


Additional file Figure 4. Association between density of fitness centers and incident CVD in 500-m buffer.

Model 1: not adjusted.

Model 2: adjusted by age, sex, education, occupation, household income, smoking, current drinking, body mass index, systolic blood pressure, fasting blood glucose, total cholesterol, antihypertensive treatment, glucose-lowering treatment, statin treatment, and population density.

Model 3: adjusted for covariates in Model 2 plus additional adjustment for leisure-time physical activity.

Abbreviations: CHD, coronary heart disease; CI, confidence interval; CVD, cardiovascular disease; HR, hazard ratio; N, number of participants; No., number.


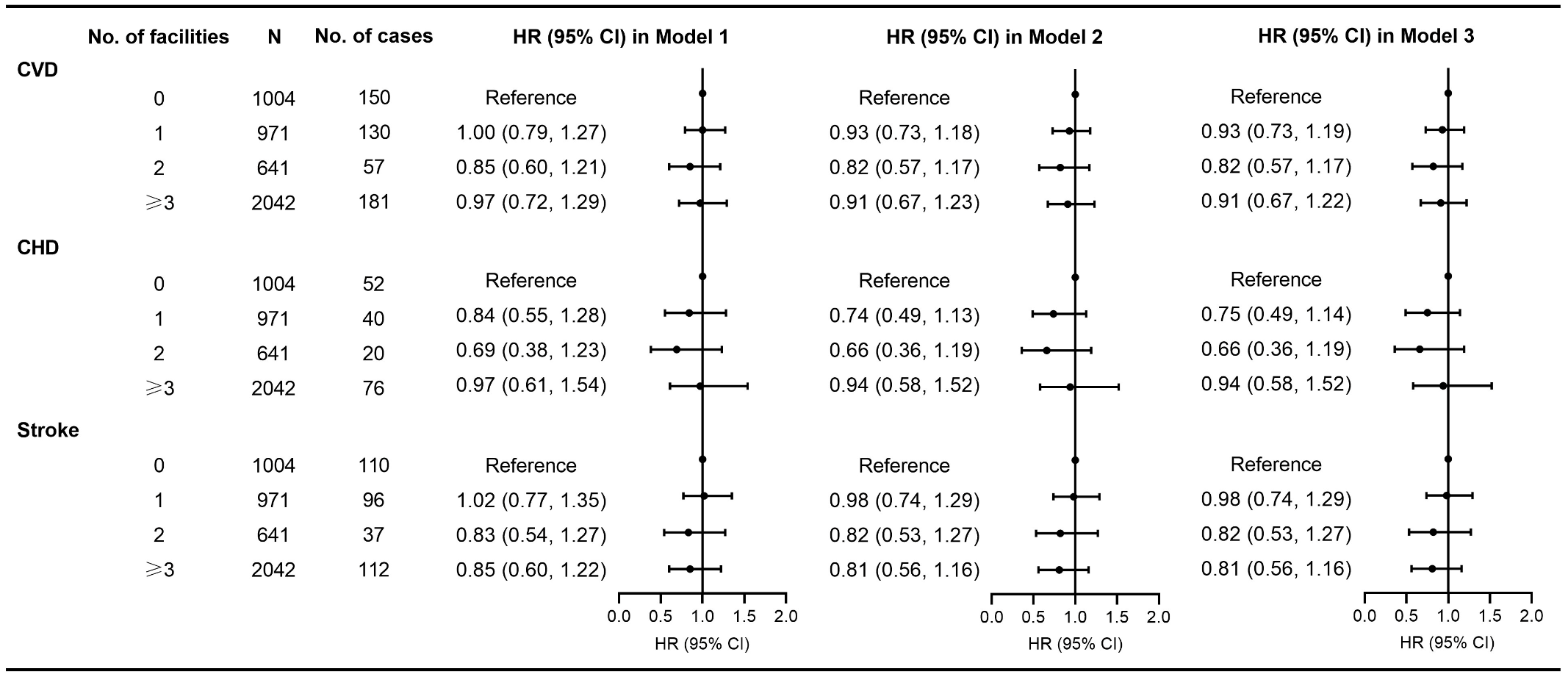


Additional file Figure 5. Association between density of physical activity facilities in 500-m buffer and incident CVD using shared frailty models.

Model 1: not adjusted.

Model 2: adjusted by age, sex, education, occupation, household income, smoking, current drinking, body mass index, systolic blood pressure, fasting blood glucose, total cholesterol, antihypertensive treatment, glucose-lowering treatment, statin treatment, and population density.

Model 3: adjusted for covariates in Model 2 plus additional adjustment for leisure-time physical activity.

Abbreviations: CHD, coronary heart disease; CI, confidence interval; CVD, cardiovascular disease; HR, hazard ratio; N, number of participants; No., number.

1. **Additional file Tables**

Additional file Table 1. Mediation analyses of the association between the presence of physical activity facilities in 500-m buffer and incident CVD through leisure-time physical activity.

|  | Total effect  β (95% CI) | *P*-value | Direct effect  β (95% CI) | *P*-value | Indirect effect  β (95% CI) | *P*-value |
| --- | --- | --- | --- | --- | --- | --- |
| CVD |  |  |  |  |  |  |
|  | -0.022 (-0.046, 0.00) | 0.042 | -0.021 (-0.045, 0.00) | 0.044 | -0.001 (-0.002, 0.00) | 0.146 |
| Stroke |  |  |  |  |  |  |
|  | -0.019 (-0.040, 0.00) | 0.038 | -0.019 (-0.040, 0.00) | 0.042 | -0.00004 (-0.00123, 0.00) | 0.926 |

Models were adjusted for age, sex, education, occupation, household income, smoking, current drinking, body mass index, systolic blood pressure, fasting blood glucose, total cholesterol, antihypertensive treatment, glucose-lowering treatment, statin treatment, and population density.

Abbreviations: CVD, cardiovascular disease.

Additional file Table 2. Association between the availability of physical activity facilities in 500-m buffer and incident CVD after additionally adjusting for distance to the major road from each participant’s residence.

| Availability of PA facilities | CVD | CHD | Stroke |
| --- | --- | --- | --- |
| Presence of PA facilities |  |  |  |
| No | Reference | Reference | Reference |
| Yes | 0.81 (0.65, 0.99) | 0.80 (0.56, 1.14) | 0.78 (0.60, 1.00) |
| Density (number) of PA facilities |  |  |  |
| 0 | Reference | Reference | Reference |
| 1 | 0.95 (0.75, 1.22) | 0.80 (0.52, 1.23) | 0.99 (0.74, 1.32) |
| 2 | 0.65 (0.47, 0.91) | 0.64 (0.37, 1.13) | 0.61 (0.41, 0.91) |
| ≥3 | 0.73 (0.56, 0.94) | 0.86 (0.56, 1.32) | 0.64 (0.47, 0.87) |

Hazard ratios and 95% [confidence interval](javascript:;)s were calculated after adjustments for age, sex, education, occupation, household income, smoking, current drinking, body mass index, [systolic](javascript:;) blood [pressure](javascript:;), fasting blood glucose, total cholesterol, antihypertensive treatment, glucose-lowering treatment, statin treatment, population density, leisure-time physical activity, and distance to the major road from each participant’s residence.

Abbreviations: CHD, coronary heart disease; CVD, cardiovascular disease; PA, physical activity.

Additional file Table 3. Association between presence of fitness centers in 500-m buffer and incident CVD.

|  | N | No. of cases | Model 1  HR (95% CI) | Model 2  HR (95% CI) | Model 3  HR (95% CI) |
| --- | --- | --- | --- | --- | --- |
| CVD |  |  |  |  |  |
| No fitness centers | 1217 | 171 | Reference | Reference | Reference |
| Presence of fitness centers | 3441 | 347 | 0.64 (0.53, 0.77) | 0.81 (0.66, 0.98) | 0.81 (0.67, 0.99) |
| CHD |  |  |  |  |  |
| No fitness centers | 1217 | 58 | Reference | Reference | Reference |
| Presence of fitness centers | 3441 | 130 | 0.70 (0.51, 0.95) | 0.81 (0.58, 1.14) | 0.83 (0.59, 1.16) |
| Stroke |  |  |  |  |  |
| No fitness centers | 1217 | 127 | Reference | Reference | Reference |
| Presence of fitness centers | 3441 | 228 | 0.57 (0.46, 0.71) | 0.75 (0.59, 0.94) | 0.75 (0.59, 0.95) |

Model 1: not adjusted.

Model 2: adjusted by age, sex, education, occupation, household income, smoking, current drinking, body mass index, [systolic](javascript:;) blood [pressure](javascript:;), fasting blood glucose, total cholesterol, antihypertensive treatment, glucose-lowering treatment, statin treatment, and population density.

Model 3: adjusted for covariates in Model 2 plus additional adjustment for leisure-time physical activity.

Abbreviations: CHD, coronary heart disease; CI, [confidence interval](javascript:;); CVD, cardiovascular disease; HR, hazard ratio; N, number of participants; No., number.

Additional file Table 4. Association between presence of physical activity facilities in 500-m buffer and incident CVD using shared frailty models.

|  | N | No. of cases | Model 1  HR (95% CI) | Model 2  HR (95% CI) | Model 3  HR (95% CI) |
| --- | --- | --- | --- | --- | --- |
| CVD |  |  |  |  |  |
| No PA facilities | 1004 | 150 | Reference | Reference | Reference |
| Presence of PA facilities | 3654 | 368 | 0.98 (0.78, 1.22) | 0.91 (0.73, 1.14) | 0.91 (0.73, 1.14) |
| CHD |  |  |  |  |  |
| No PA facilities | 1004 | 52 | Reference | Reference | Reference |
| Presence of PA facilities | 3654 | 136 | 0.85 (0.59, 1.24) | 0.78 (0.54, 1.13) | 0.79 (0.54, 1.14) |
| Stroke |  |  |  |  |  |
| No PA facilities | 1004 | 110 | Reference | Reference | Reference |
| Presence of PA facilities | 3654 | 245 | 0.96 (0.74, 1.25) | 0.92 (0.71, 1.20) | 0.92 (0.71, 1.20) |

Model 1: not adjusted.

Model 2: adjusted by age, sex, education, occupation, household income, smoking, current drinking, body mass index, [systolic](javascript:;) blood [pressure](javascript:;), fasting blood glucose, total cholesterol, antihypertensive treatment, glucose-lowering treatment, statin treatment, and population density.

Model 3: adjusted for covariates in Model 2 plus additional adjustment for leisure-time physical activity.

Abbreviations: CHD, coronary heart disease; CI, [confidence interval](javascript:;); CVD, cardiovascular disease; HR, hazard ratio; N, number of participants; No., number; PA, physical activity.
